# Ectopic Cushing' syndrome caused by a neuroendocrine carcinoma of the mesentery

**DOI:** 10.1186/1471-2407-6-108

**Published:** 2006-04-27

**Authors:** Mathias Fasshauer, Thomas Lincke, Helmut Witzigmann, Regine Kluge, Andrea Tannapfel, Michael Moche, Michael Buchfelder, Stephan Petersenn, Juergen Kratzsch, Ralf Paschke, Christian A Koch

**Affiliations:** 1 Division of Endocrinology and Nephrology, University of Leipzig, Philipp-Rosenthalstr. 27, 04103 Leipzig, Germany; 2 Department of Nuclear Medicine, University of Leipzig, Liebigstr., 04103 Leipzig, Germany; 3 Department of Surgery, University of Leipzig, Liebigstr., 04103 Leipzig, Germany; 4 Institute of Pathology, Ruhr-Universität Bochum an den BG Kliniken Bergmannsheil, Bürkle-de-la-Camp-Platz 1, 44 789 Bochum, Germany; 5 Department of Radiology, University of Leipzig, Liebigstr., 04103 Leipzig, Germany; 6 Department of Neurosurgery, Friedrich-Alexander University of Erlangen-Nuremberg, Schwabachanlage, Erlangen, Germany; 7 Division of Endocrinology, Universität Duisburg-Essen, Hufelandstr. 55, 45122 Essen, Germany; 8 Institute of Laboratory Medicine, Clinical Chemistry, and Molecular Diagnostics, University of Leipzig, Paul-List-Str., 04103 Leipzig, Germany; 9 Division of Endocrinology, University of Mississippi Medical Center, 2500 N State Str, Jackson, MS 39216, USA

## Abstract

**Background:**

ACTH overproduction within the pituitary gland or ectopically leads to hypercortisolism. Here, we report the first case of Cushing' syndrome caused by an ectopic ACTH-secreting neuroendocrine carcinoma of the mesentery. Moreover, diagnostic procedures and pitfalls associated with ectopic ACTH-secreting tumors are demonstrated and discussed.

**Case presentation:**

A 41 year-old man presented with clinical features and biochemical tests suggestive of ectopic Cushing's syndrome. First, subtotal thyroidectomy was performed without remission of hypercortisolism, because an octreotide scan showed increased activity in the left thyroid gland and an ultrasound revealed nodules in both thyroid lobes one of which was autonomous. In addition, the patient had a 3 mm hypoenhancing lesion of the neurohypophysis and a 1 cm large adrenal tumor. Surgical removal of the pituitary lesion within the posterior lobe did not improve hypercortisolism and we continued to treat the patient with metyrapone to block cortisol production. At 18-months follow-up from initial presentation, we detected an ACTH-producing neuroendocrine carcinoma of the mesentery by using a combination of octreotide scan, computed tomography scan, and positron emission tomography. Intraoperatively, use of a gamma probe after administration of radiolabeled ^111^In-pentetreotide helped identify the mesenteric neuroendocrine tumor. After removal of this carcinoma, the patient improved clinically. Laboratory testing confirmed remission of hypercortisolism. An octreotide scan 7 months after surgery showed normal results.

**Conclusion:**

This case underscores the diagnostic challenge in identifying an ectopic ACTH-producing tumor and the pluripotency of cells, in this case of mesenteric cells that can start producing and secreting ACTH. It thereby helps elucidate the pathogenesis of neuroendocrine tumors. This case also suggests that patients with ectopic Cushing's syndrome and an octreotide scan positive in atypical locations may benefit from explorative radioguided surgery using ^111^In-pentetreotide and a gamma probe.

## Background

Endocrine tumors can develop from a variety of tissue types and are often under- or misdiagnosed which underscores the difficulties to classify them. Ectopic ACTH overproduction not only leads to hypercortisolism but is also associated with various diagnostic and therapeutic pitfalls despite performing extensive diagnostic procedures.

## Case presentation

**Figure 1:**
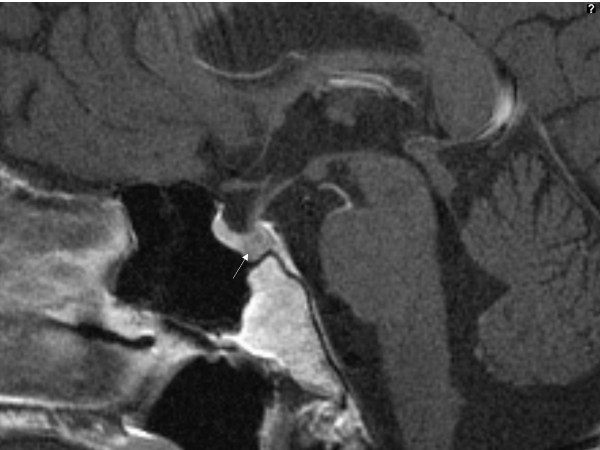
**T1-weighted gadolinium-enhanced MRI of the pituitary**. A hypointense lesion of the neurohypophysis is indicated.

**Figure 2:**
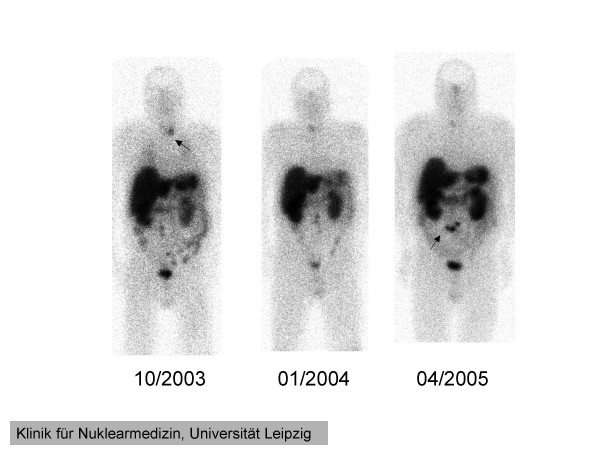
**^111^In-pentetreotide scintigraphy**. Radiotracer accumulation in the left thyroid in 10/2003 (arrow). The mesenterial neuroendocrine tumor became clearly visible in 4/2005 (arrow).

In September 2003, a 41-year-old man presented with unexplained progressive weight gain, facial plethora, tremor, sweating, newly-diagnosed hypertension, hypokalemia, and diabetes mellitus. Physical examination revealed a blood pressure of 170/110 mm Hg, pulse of 88 bpm, thyroid enlargement, and cushingoid features including striae, moon face, central obesity, and a buffalo hump. Laboratory testing showed hyperthyroidism (undetectable TSH, free T4 of 32 pmol/L (normal: 12–22), free T3 of 9 pmol/L (normal: 3.95–6.80)) and increased 24 h urinary free cortisol (7579 nmol per day, normal range: 22–212). Thyroid ultrasound showed a multinodular goiter. A Tc-99m-pertechnetate thyroid scan revealed increased uptake in an area of the left thyroid gland corresponding to an autonomous adenoma. Serum calcitonin was within the normal range. Basal serum cortisol and plasma ACTH were significantly elevated at 1307 nmol/l (normal range: 187–724) and 22.51 pmol/l (1.98–11.4), respectively. After a 2-mg overnight dexamethasone suppression test, serum cortisol was still at 1132 nmol/l (normal, < 80). Midnight cortisol was elevated at 847 nmol/l. After an overnight 8-mg dexamethasone suppression test, serum cortisol decreased by 38 % of basal values suggesting ectopic ACTH-secretion (EAS). CRH stimulated serum cortisol and plasma ACTH to less than 4 % of basal values. Inferior petrosal sinus sampling (IPSS) did not show significant central-to-peripheral ACTH gradients. Ectopic ACTH syndrome was diagnosed. Computed tomography (CT) scans at first presentation did not show pulmonary and/or abdominal tumors, however, both adrenals appeared enlarged with the left gland showing a tumor of 1 cm size. A magnetic resonance imaging (MRI) scan of the head demonstrated a small 3 mm hypointense and hypoenhancing lesion of the neurohypophysis (Fig. 1). An ^111^In-pentetreotide scan showed radiotracer enrichment in the left thyroid gland consistent with the region of the autonomous adenoma and faint focal octreotide enhancement in the abdomen which was interpreted to be nonspecific because of lacking correlation on CT scan and changing location of enhancement between early and late scan (Fig. 2). Positron emission tomography (PET) with ^18^F- fluorodeoxyglucose (FDG) and colonoscopy showed normal results. Thyroid antibodies were negative, supporting the scintigraphic finding that hyperthyroidism in this patient was caused by an autonomous adenoma. Given the scenario of EAS and an octreoscan being reliably positive only in the left thyroid gland, we decided to perform a left-sided hemithyroidectomy and subtotal resection of the right gland after treatment with methimazole, although basal calcitonin was normal and pentagastrin stimulation could not be performed because it was not available. Extensive pathological workup of the thyroid specimen did not reveal an ACTH-producing endocrine tumor but a 2 mm sized benign nodule on the right side, and 2 benign nodules (1 cm and 2 cm in diameter, respectively) within the left thyroid gland.

**Figure 3:**
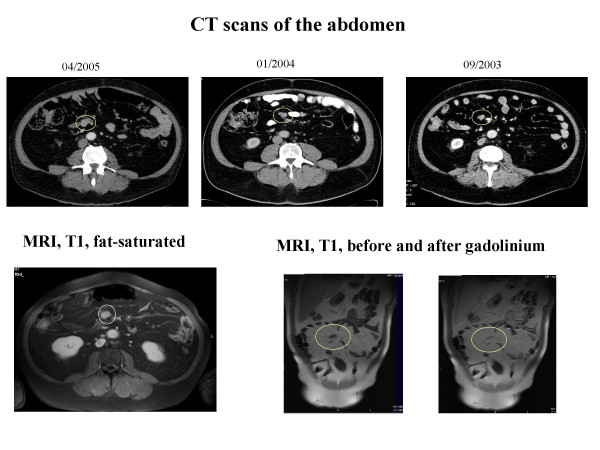
**CT and MRI scans of the abdomen**. In 4/2005, a mass (circle) is shown which has also been there in earlier scans but had been interpreted as normal intestine. Fat-saturated and T1-weighted MRI scanning after gadolinium shows a mass in the mesentery outside the intestine.

**Figure 4:**
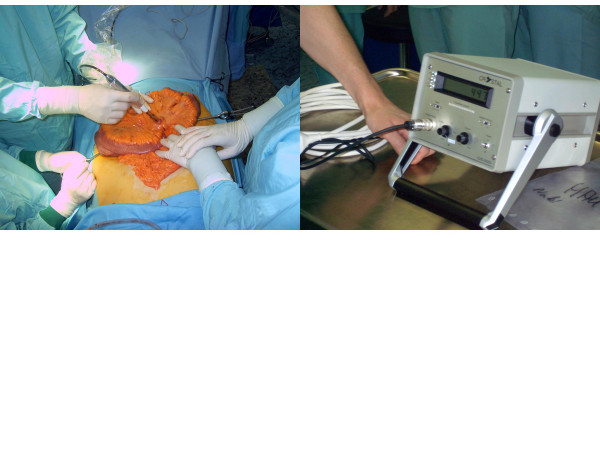
Gamma probe used intraoperatively.

**Figure 5:**
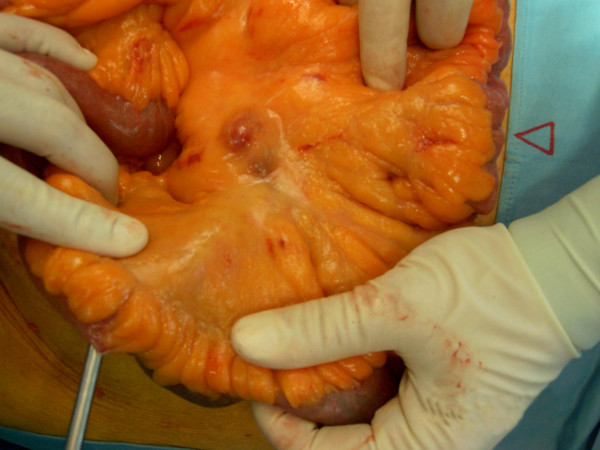
**Intraoperative situs**. A brown tumor mass is visible within the yellow mesentery.

**Figure 6:**
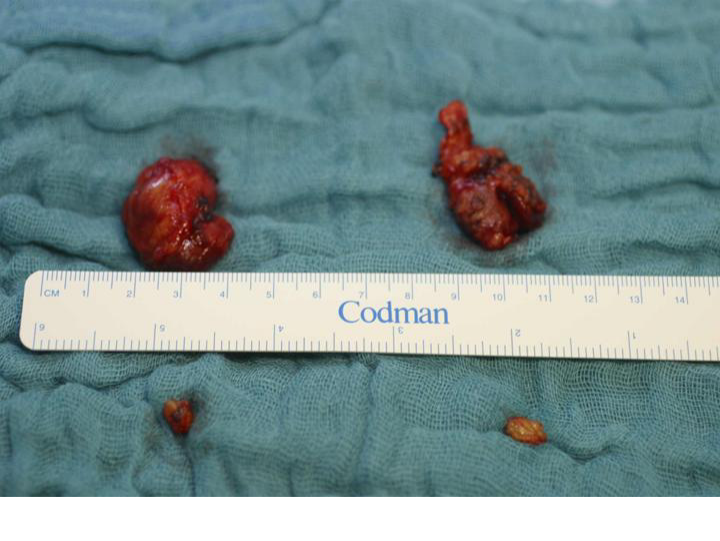
Macroscopic image of the tumor and removed lymph nodes.

**Figure 7:**
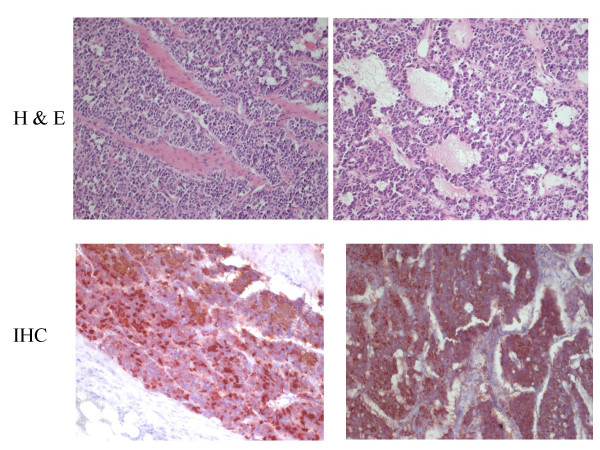
Images of histopathological Hematoxylin and Eosin stains and immunohistochemical ACTH expression.

Whereas hyperthyroidism improved rapidly, hypercortisolism persisted. Because the patient had elevated liver function tests, thereby limiting the use of ketoconazole, we began metyrapone (2.5 g/d divided into 750 mg in the morning and at noon, and 1 g in the evening to perform medical adrenalectomy) combined with hydrocortisone replacement (10 mg in the morning at at noon, and 5 mg in the evening) to achieve normal 24 h urinary cortisol values. Regular follow-up examinations at 3, 6, 9, and 12 months after initial presentation confirmed the pituitary lesion within the posterior lobe, while CT scans of thorax and abdomen remained negative except for the left-sided adrenal tumor. 24 h urinary catecholamines including fractionated metanephrines were negative. Over the following months, control of hypercortisolism by metyrapone became difficult and the question arose whether bilateral adrenalectomy should be performed. Therefore, we consulted a neuropathologist/pathologist (A.O.V.) and neurosurgeon (E.H.O.) at the National Institutes of Health whether they ever had seen an ACTH-producing pituitary adenoma located in the posterior lobe (see ref. 1). Subsequently, transsphenoidal surgery was performed to explore the pituitary lesion in our patient. Despite complete removal of this lesion, hypercortisolism persisted and metyrapone therapy was continued. Histological workup of the removed pituitary tissue revealed normal posterior pituitary. Postoperatively, diabetes insipidus developed and persisted. An extensive re-evaluation was performed 18 months after initial presentation. An octreoscan revealed tracer accumulation in projection of the upper and middle part of the right abdomen (Fig. 2). Additional ^18^F-FDG PET showed increased glucose metabolism of this abdominal area. In accordance with these findings, a 21 × 15 mm tumor mass was demonstrated on CT and MRI scans in this region (Fig. 3). In retrospect, this tumor could also be seen in earlier CT scans but had been interpreted as normal intestine because of smaller size, atypical location for an ACTH-producing tumor, and only moderate uptake on octreotide scans. We decided to perform an explorative laparotomy. Before surgery, ^111^In-pentetreotide was administered to support tumor localization during surgery with a gamma counter and facilitate maximal tumor removal (Fig. 4). After the abdominal wall had been opened, the neuroendocrine tumor was instantly visible (Fig. 5). Because this lesion had been initially assumed to be metastatic tissue of a primary neuroendocrine tumor and a multifocal appearance of the abdominal ^111^In-pentetreotide enhancement, the gamma probe was used to detect further tumor tissue (Fig. 6). Histopathological and immunohistochemical analyses of the removed tissue revealed a well-differentiated neuroendocrine carcinoma of the mesentery with strong expression of ACTH (Fig. 7). The tumor also strongly expressed NSE, chromogranin A, and synaptophysin. The MIB-1 proliferation index was approximately 1 %. Immunohistochemical analyses for the expression of somatostatin receptors type 1 to 5 were negative. All removed lymph nodes were tumor-negative but also showed increased activity by the gamma probe during surgery.

Follow-up at four weeks and at 4 (7) months after surgery showed a normal 2 mg dexamethasone suppression test and 24 h urinary cortisol levels. Plasma ACTH levels were normal (4.9 and 5.2 pmol/L, normal: 1.8–12.8). NSE was 10.7 ng/ml (normal, < 13), chromogranin A and calcitonin were undetectable. In addition, the insulin requirement to treat the patient' s type 2 diabetes declined by more than 50%. At 7 month follow-up, the patient had regained strength and muscle mass, did not need further potassium replacement, and felt fine. An octreoscan showed normal results.

## Discussion

We present for the first time a case of EAS caused by a neuroendocrine carcinoma of the mesentery. Among ectopic ACTH-producing lesions, approximately 50% originate from intrathoracic tumors, usually small cell carcinomas [2-6]. Other well-described tumors of ectopic ACTH-production include medullary thyroid carcinoma, pheochromocytoma, as well as carcinomas of the thymus and pancreas [2,7-11]. Except for the pancreas, other organs and locations for ACTH-producing tumors in the abdomen, i.e. appendix, duodenum, ileum, colon, anal canal, uterine cervix, and ovary are uncommon [12-20]. Endocrine tumors can develop from a variety of tissue types and are often under- or misdiagnosed which underscores the difficulties to classify them [21]. Endocrine tumors may or may not produce and/or secrete hormones [22-32]. Mesenchymal tumors such as solitary fibrous tumors of the pleura, for instance, can lead to tumor hypoglycemia through production and secretion of pro-IGF-II [Fasshauer and Koch, unpublished observation; [33,34]]. This ability of tumor cells making hormones is not surprising, if one considers the embryologic origin of tissues. For instance, upon induction, ectoderm of the blastula differentiates into mesodermal tissue such as chorda, muscle, and fibrous tissue. Without induction, however, ectoderm will differentiate into epidermis-like epithelium [35]. Arita-Melzer et al. [24] reported a 76-yo man who suffered from Cushing's syndrome caused by ectopic ACTH production of a sacrococcygeal chordoma. In our patient, we propose that cells of the mesentery became malignant and started to produce ACTH ectopically. Although all removed lymph nodes were negative for tumor and the patient postoperatively became and remained eucortisolemic at 7 month follow-up, there is still a small chance that he harbors somewhere else a primary endocrine tumor producing ACTH that had metastasized to the mesentery. Another atypical location for primary endocrine tumors that had been controversially discussed for a long time is the liver. Maire et al. [36] has recently shown that this organ can harbor primary endocrine tumors, although the histogenesis of these tumors remains unknown. One hypothesis claims that pluripotent stem cells in the liver lead to primary endocrine tumors, a theory that is supported by a study showing that rat hepatic stem cells can differentiate into pancreatic functional endocrine cells [37]. Another hypothesis assumes that these liver tumor cells are of neuroectodermal origin. In our patient, no liver lesions were seen. Primary endocrine tumors have also been reported in lymph nodes [38]. In our patient, however, the removed tumor tissue and lymph nodes did not demonstrate evidence for lymph nodes as an origin of tumor growth.

Diagnosing EAS is often difficult [39,40]. None of the dynamic biochemical tests achieves 100% accuracy, although BIPSS almost always shows an absent central gradient [41]. A recent report by Ilias and co-workers emphasizes that 21 % and 26 % of patients with EAS have false-positive responses to dexamethasone and/or CRH [6]. In their hands, IPSS is the single best test for EAS that correctly identified 66 out of 67 patients [6]. In our case, all three independent tests suggested EAS, since a) the decline of serum cortisol was less than 50 % in the overnight 8-mg dexamethasone suppression test as compared to basal values, b) the increase in serum cortisol and plasma ACTH was less than 20 % and 35 %, respectively, after CRH stimulation, and c) IPSS demonstrated a less than 2-fold central-to-peripheral ACTH gradient basal and less than 3-fold gradient after CRH administration.

However, in the case presented, correct localization of tumor causing the EAS had been difficult. The percentage of tumors not identified despite extensive evaluation is between 12% and 19% [5,6,42]. In our case, imaging studies consisted of CT scans of chest, abdomen, and pelvis, an MRI scan of the pituitary, octreotide scans, and ^18^F-FDG PET at initial presentation and during follow-up. Except for PET, these tests are commonly used to locate the cause of EAS [2,39,43-47]. Furthermore, octreotide has been suggested not only as a diagnostic tool but also as second-line therapy in some cases of EAS [48,49]. PET is a second-line diagnostic procedure when CT scan, MRI, and octreotide scan do not locate the cause of EAS [45]. In the case presented, only the octreotide scan and the MRI of the pituitary had yielded pathological results at first presentation. Since EAS has been described in medullary thyroid carcinoma and the patient had multinodular goiter in association with hyperthyroidism, subtotal thyroidectomy was performed with the intention to exclude medullary thyroid carcinoma and resect the proven autonomous adenoma simultaneously in the face of a normal basal calcitonin and lacking pentagastrin stimulated calcitonin [8,10]. However, this surgical procedure did not improve EAS. This result supports recent findings by Becker and co-workers that false-positive octreotide scans occur in endemic goiter with or without thyroid autonomy [50]. Furthermore, it confirms recent reports indicating that a positive octreotide scan alone may not be sufficient to locate EAS [51]. In our patient, the octreotide scan was also weakly positive in an abdominal area not very typical for an ACTH-producing endocrine tumor that later on, however, proved to be the cause for EAS. Similarly, an ^111^In-pentetreotide scan provided the decisive clue for locating a malignant ACTH-producing tumor of the ileum in a patient reported by Segu et al. [15]. This suggests that patients with EAS and a positive octreoscan in locations atypical for an ACTH-producing tumor may benefit from explorative surgery using ^111^In-pentetreotide and a gamma probe.

Complicating the management of our patient with EAS and clearcut negative IPSS, a small lesion of the neurohypophysis was demonstrated at first presentation and during 1-year follow-up. False-negative results of IPPS in patients who do have an ACTH-producing pituitary tumor are reported to occur in up to 13% (7/127) [52]. Given the increasingly difficult to control hypercortisolemia, the low risk of transsphenoidal surgery, and the recent findings of clinical researchers at the National Institutes of Health, where surgical removal of ACTHomas confined to the neurohypophysis resulted in remission of hypercortisolism in 12 patients [1], we decided to proceed with an exploration of the pituitary. However, in our case removal of the posterior lobe pituitary lesion did not result in remission of hypercortisolemia. These results re-emphasize the notion that a single positive imaging study may represent a falsely positive result, while more than one positive study in the same region may confirm a true ACTH-secreting lesion [2,6]. It also underscores that in patients with Cushing's syndrome, dynamic biochemical testing is the most important step in establishing the diagnosis. Because our patient with EAS also had a left sided adrenal tumor in the setting of bilaterally enlarged adrenal glands, we measured 24 h urinary catecholamines including metanephrines to exclude an ACTH-producing pheochromocytoma which can occur in up to 25% of patients with EAS. In a recent study, 3 of 28 patients with ACTH-dependent Cushing's syndrome had focal adrenal nodules [53]. Although non-catecholamine secreting pheochromocytomas have been described [9,54], this is unlikely in our patient who had remission of hypercortisolism and ACTH production after the mesenterial tumor had been removed.

At 18-months after first presentation, another extensive diagnostic procedure was performed including CT scans of chest, abdomen, and pelvis, MRI head, octreotide scan, and ^18^F-FDG PET. Here, tracer accumulation in projection of the upper and middle part of the right abdomen was detectable in the octreotide scan and PET, for the first time corresponding to a 21 × 15 mm and 26 × 15 mm tumor mass on CT and MRI scans, respectively. These results support the view that regular follow-up of occult EAS may result in localization of ACTH production at later time points due to tumor growth [2,6]. Furthermore, we could show that application of ^111^In-pentetreotide within hours before surgery facilitates localization of the tumor during surgery by use of radiolabeled ^111^In-pentetreotide and a gamma probe similar to recent reports [55,56].

To further elucidate whether patients with neuroendocrine tumors that are (supposedly) completely resected, may benefit from adjuvant therapy including somatostatin analogs, we performed a MIB-1 proliferation index of the tumor in our patient and immunohistochemical analyses of somatostatin receptor expression [57,58]. We found all 5 somatostatin receptors negative using immunohistochemistry [59,60]. Especially the negative results for somatostatin receptor type 2 stand in contrast to our results on the octreoscans, although the tumor exceeded a size of 5 mm which is usually regarded as the detection limit for an octreoscan. These results may indicate that this ACTH-producing neuroendocrine tumor is very heterogeneous and/or somatostatin receptors were downregulated/saturated by intraoperative exposure to high doses of ^111^In-pentetreotide. Unfortunately, we were not able to assess mRNA for somatostatin receptor expression.

## Conclusion

We report for the first time a patient with EAS due to a neuroendocrine carcinoma of the mesentery. This case not only may help elucidate the pathogenesis of (ACTH-producing) endocrine tumors, but also illustrates diagnostic pitfalls in the management of patients with EAS, and suggests that patients with EAS and an octreoscan positive in atypical locations may benefit from explorative radioguided surgery using a gamma probe.

## Abbreviations

CT – computed tomography; EAS – ectopic ACTH-secretion; FDG – fluorodeoxyglucose; IPSS – inferior petrosal sinus sampling; MRI – magnetic resonance imaging; PET – positron emission tomography

## Competing interests

Novartis Pharma GmbH will carry the costs of the article-processing charge in conjunction with a grant provided to Prof. Koch (CAK) on the topic "Neuroendocrine tumors: Nachuntersuchung aller operierten (R0/komplett resezierten) Patienten mit gastroenteropankreatischen neuroendokrinen Tumoren mittels Octreoscan". The authors declare that they have no other competing interests.

## Authors' contributions

MF provided clinical care for this patient and drafted the paper. TL provided clinical care for this patient and helped draft and interpret the results, especially of studies related to nuclear medicine. HW provided clinical care for this patient and operated on the patient. RK provided clinical care for this patient and helped draft and interpret the results, especially of studies related to nuclear medicine. AT provided important insights into the histopathological diagnosis of this patient, delivered macroscopic, microscopic, and immunohistochemical images of this patient's tumor, and helped interpret the results. MM provided clinical care for this patient and helped interpret radiological images performed in this patient. MB conducted transsphenoidal surgery in this patient and helped interpret the clinical course and results of various tests. SP carried out immunohistochemical studies on somatostatin receptor types and helped interpret the results and draft the paper. JK delivered important insights into the laboratory and clinical chemistry aspects of this patient's history. RP helped interpret the results of all studies performed in this patient and provided intellectual input. CAK designed this study, interpreted the results, drafted the paper, and finalized the manuscript after input from the other authors.

All authors read and approved the final version of the manuscript.

## Pre-publication history

The pre-publication history for this paper can be accessed here:
